# Self‐microemulsifying oral fast dissolving films of vitamin D3 for infants: Preparation and characterization

**DOI:** 10.1002/fsn3.1108

**Published:** 2019-07-11

**Authors:** Min Zhang, Tingrui Zhang, Ying Zou, Ping Han, Kehai Liu

**Affiliations:** ^1^ College of Food Science and Technology Shanghai Ocean University Shanghai China; ^2^ National Experimental Teaching Demonstration Center for Food Science and Engineering Shanghai Ocean University Shanghai China

**Keywords:** infant, oral fast dissolving films, release kinetics, self‐microemulsifying, vitamin D

## Abstract

Combining the advantages of self‐microemulsifying technology and oral fast dissolving technology, a self‐microemulsifying oral fast dissolving films (SMEOFDF) of vitamin D3 was developed in this study. The pseudoternary phase diagram of microemulsion was constructed using water titration method, and the formulation of films was optimized by orthogonal experimental design. The prepared SMEOFDF of vitamin D3 was a thin film, in which the liquid drops of self‐microemulsion were embedded. It had good mechanical properties (thickness 166.7 ± 3.30 µm, tensile strength 38.45 ± 3.72 MPa, elongation 23.38 ± 4.23%, and folding endurance >200 times), and its disintegration time was about 18 ± 1.23 s. After being redissolved in water, microemulsion could form spontaneously, with particle size of 181.2 nm and zeta potential of 16.1 mV. The release profile of vitamin D from SMEOFDF could be well described by first‐order equation.

## 1 INTRODUCTION

Infants and young children are at high risk of micronutrient deficiency, and nutritional supplements play a vital role in safeguarding growth and promoting health. There are differences in the requirement of nutritional supplements at different ages, with vitamin D supplementation most common throughout infancy and young children. Vitamin D is important in musculoskeletal development and calcium homeostasis (Institute of Medicine, 2011). Not only poor vitamin D status may lead to rickets in infants and young children, but also more recent evidence which links vitamin D to various nonskeletal disorders including lung dysfunction, disordered immune response, and suboptimal growth (Thiele, Ralph, El‐Masri, & Anderson, 2017). As daily intake of vitamin D3 in infants, 400 IU is recommended by the American Academy of Pediatrics currently (Wagner & Greer, 2008).

Vitamins, as a chemically heterogeneous class of compounds, are classified into water‐soluble and fat‐soluble vitamins. Vitamin D is a fat‐soluble and water‐insoluble substance, which has the disadvantages of slow dissolution and poor absorption, and its oral bioavailability is generally low (El‐Sherbiny et al., 2018). On the other hand，oral administration is the main administration route, accounts for the majority of total treatment (Lajoinie et al., 2016). Currently, most oral dosage forms of nutrition supplements for infants and young children are granules, tablets, and other solid preparations as well as liquid preparations such as oral liquid, drops, and syrups. However, these dosage forms have some disadvantages for infants and young children. Solid preparations have difficulty swallowing. Liquid preparations are prone to cough and choke. Also, it is easy to lose when taken, resulting in inaccurate dosage (Ranmal, Cram, & Tuleu, 2016; Walsh, Ranmal, Ernest, & Liu, 2018). At the same time, because the neurodevelopment of children is not perfect, there will be the psychology and behavior of rejecting drugs with special taste or irritating smell. Therefore, the oral administration of drugs to infants is a difficult process. Sometimes, when the child cries, the full amount of medicine will be poured into the mouth quickly, easily causing the child cough, vomiting, inhalation pneumonia, and other injuries. Therefore, oral administration for infants and young children not only causes a certain degree of drug waste and affects the expected treatment effect, but also increases the psychological burden on their parents, further aggravates the psychological damage of infants, and makes them more fearful of oral administration.

Based on the above analysis, the above two problems can be solved by making nutrition supplements for infants and young children into the self‐microemulsifying oral fast dissolving films (SMEOFDF). As a new dosage form, SMEOFDF combines the oral fast dissolving technology with the self‐microemulsifying technology, which has both advantages. As long as a stamp‐sized thin film is put into the mouth of infants, it can be rapidly dissolved by saliva, without drinking water and swallowing, and will not cause infants choke cough (Chonkar et al., 2016; Lai et al., 2015; Shimoda et al., 2009). At the same time, it is convenient to give medicine, and the size and quantity of the films can be determined freely, so as to achieve the purpose of accurate dosage. After the films are dissolved in the mouth, self‐microemulsion with particle size of 100–1,000 nm can be formed spontaneously. Self‐microemulsifying drug delivery system (SMEDDS) is a nanodispersion system composed of oil phase, emulsifier, and co‐emulsifier. The main feature of SMEDDS is that it can automatically form microemulsion under physiological conditions. SMEDDS can improve the solubility of fat‐soluble drugs and increase the absorption rate, thus overcoming the disadvantages of low oral bioavailability of fat‐soluble drugs (such as vitamin D3; Chow, Gue, Leow, & Goh, 2015; Krstić, Medarević, Đuriš, & Ibrić, 2018; Singh et al., 2016).

Combining the advantages of self‐microemulsifying drug delivery system and fast dissolving films, a self‐microemulsifying oral fast dissolving films of vitamin D3 with advantages of convenience, quick starting, and good compliance was developed in this study, which could well solve the problems: difficulty of oral administration for infants and low oral bioavailability of fat‐soluble drug (such as vitamin D). There are no reports of making vitamin D into self‐microemulsifying oral fast dissolving films currently. In addition, SMEOFDF is suitable for large‐scale industrial production with low cost, short production cycle, strong operability, and no pollution.

## 2 MATERIALS AND METHODS

### 2.1 Materials

Methylcellulose, glycerin, lauric acid, Tween‐80, and vitamin D3 of food grade were purchased from China Pharmaceutical (Group) Company of Shanghai Chemical Reagent Corporation. Orange fruit powder was obtained from Shanghai Apple Flavor & Fragrance Co., Ltd. N‐hexane, acetonitrile, and methanol were purchased from Sinopharm Chemical Reagent Co., Ltd.

### 2.2 Construction of pseudoternary phase diagram

Pseudoternary phase diagram was constructed using water titration method in order to locate the microemulsion region and obtain the concentration range of oil, surfactant, co‐surfactant, and water in the region (Dong et al., 2016; Zhao et al., 2014). First of all, surfactant was mixed with co‐surfactant at a weight ratio (*S*_mix_) of 10:1. A series of self‐emulsifying systems consisting of oil/surfactant/co‐surfactant were prepared with the weight ratio of oil to surfactant/co‐surfactant ranging in 9:1, 8:2, 7:3, 6:4, 5:5, 4:6, 3:7, 2:8, and 1:9. Then, the distilled water was added drop by drop into each self‐emulsifying system under magnetic stirring at 37°C, respectively. The mixture was examined visually in this process until it turned from transparent to cloudy. At last, the percentage of each component was calculated out according to the amount of water added. The pseudoternary phase diagram was drawn with OriginPro 8.5 software.

### 2.3 Preparation of SMEOFDF of vitamin D3

Lauric acid and Tween‐80 were mixed in thermostat‐controlled waterbath at 37°C as the compound emulsifier, to which a precalculated quantity of vitamin D3 was added. The solution was stirred with a magnetic stirring apparatus (DF‐101S, Qiangqiang Equipment Co.) to ensure thorough dissolution. The film‐forming solution was prepared by dissolving MC and glycerin in a certain amount of water and added with orange fruit powder. The emulsifier solution was added into the film‐forming solution, fixed the volume with water, and stirred thoroughly until completely dispersed to form the milky white mixture. The mixture was then injected into a special mold, pour film to shape. After dried at 40°C in dark, the film was cut into different sizes to get the final product (Nishigaki et al., 2012).

### 2.4 Optimization of the SMEOFDF formulation

The SMEOFDF formulation was optimized by orthogonal experiments L_9_ (3^4^) using formability and dissolution time as the target index. In the formability evaluation, sensory score (full 100 points) was conducted from the appearance and surface. Dissolution time referred to the time when the films disintegrated in water and completely disappeared. The data were analyzed by multi‐index comprehensive weighted score evaluation. The MC dosage, glycerin dosage, and compound emulsifier dosage with three levels were chosen in the orthogonal experiments (Table 1). Variance analysis and directly analyzing on the results of the orthogonal experiments were carried out. The optimum conditions were further verified by duplicate tests.

**Table 1:** Factors and levels

Levels^a^	Factors		
	MC dosage (g)	Glycerin dosage (ml)	Compound emulsifier dosage (g)
	A	B	C
1	2.0	4	1
2	2.5	8	2
3	3.0	12	3

a The dosages of MC, glycerin, and compound emulsifier refer to the amount contained in the 100 ml mixture.

### 2.5 Investigation of particle size, zeta potential, and morphology

The structural and morphological analysis is an important characterization of the films. The morphology and microstructure of SMEOFDF were observed by inverted microscopy (AE‐31, Motic Corporation) and transmission electron microscopy (JEM 2100F, JEOL Ltd.), respectively.

Microemulsion is formed spontaneously after the dissolution of SMEOFDF, and the particle size is directly related to its solubility and bioavailability. Therefore, it is of great significance for the detection and control of its particle size, which would better be the range between 100 and 400 nm generally. Zeta potential is the strength measurement of mutual repulsion or attraction between microemulsion droplets, and an important parameter to measure the stability of colloidal particles. It is very important in the theory of colloidal stability, and its value is correlated with the stability of colloidal dispersion, which is generally appropriate at ±30 mV (Zhang et al., 2018). The particle sizes and zeta potentials in this study were measured using an electrophoretic light‐scattering spectrophotometer (Zetasizer Nano ZS90, MAN0317 Issue 5.0, Malvern Instruments Ltd.) with 90° scattering angles. All the experiments were performed in triplicate.

### 2.6 Test of physical properties

The thickness of the films was measured at different positions (4 angles and center) with a thickness gauge, and the mean value and standard deviation were calculated. The folding endurance was represented by the number of folds that were repeated at the same part of the membrane until it broke. SMEOFDF of vitamin D3 was cut into a standard 5 cm × 1 cm film sample, which was tested on the probe of a texture analyzer (TA.XT.PLUS, Stable Micro System Ltd.). The tensile speed was set at 25 mm/min, and the distance between the probes was 30 mm. Then, the tensile strength and elongation of the film were measured and the average value was taken for five times.

The tensile strength and elongation were calculated as follows:

$$\text{TS}\left(\text{MPa}\right)=\frac{F}{\left(L\times d\right)}$$

TS = tensile strength

*F* = the maximum tension of the sample at fracture (*N*)

*L* = film width (mm)

*d* = film thickness (mm)

$$E\left(\%\right)=\left(\frac{\left(L^{{}^{\prime }}-L\right)}{L}\right)\times 100$$

*E* = elongation

*L* = the original length (mm)

*L*′ = the elongation length (mm)

Disintegration time was noted.

The film was placed into a beaker containing 50 ml phosphate buffer pH 6.8, shaken gently at 37°C until disintegrated. Disintegration time was noted.

### 2.7 Study of in vitro release

With phosphate buffer (900 ml, pH 6.8) as the release medium, the determination of in vitro release was carried out using paddle method by dissolution tester (RCZ‐6B2, Huanghai Pharmaceutical Instrument Co.) at 37 ± 0.5°C and at paddles speed of 100 rpm; 10 ml of sample was withdrawn at different time intervals (replenished with 10 ml of fresh medium), extracted with n‐hexane, and then analyzed by HPLC (Agilent 1260, Agilent Technologies). An Alltima C_18 _column (250 mm × 4.6 mm, 5 µm) was used for the separation, with acetonitrile‐methanol (v/v = 90:10) as the mobile phase at the flow rate of 2 ml/min. The detection wavelength was 265 nm (Zhong, Xia, & Shi, 2007). In vitro release curve was drawn based on the release data. The release profile was fitted to three different kinetic models, and the goodness of fit of the release data was assessed (Verma, Rajput, & Naik, 2018).

## 3 RESULTS AND DISCUSSION

### 3.1 Pseudoternary phase diagram

**Figure 1:**
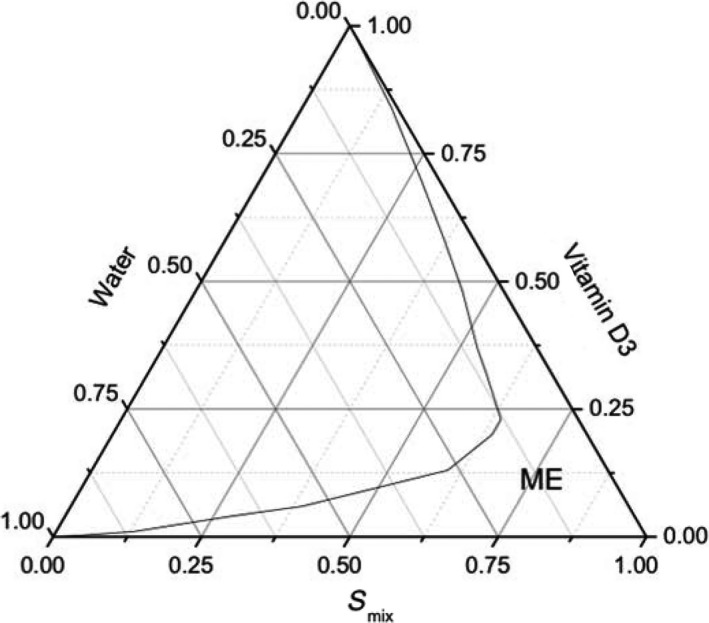
Pseudoternary phase diagram

Pseudoternary phase diagram was constructed using the water titration method, which helped to obtain the concentration range of microemulsion components and the boundary of phase transitions. In this study, a food‐grade microemulsion was presented on the pseudoternary phase diagram (Figure 1), consisted of vitamin D3 as oil phase, Tween‐80 as surfactant, lauric acid as cosurfactant, and water as aqueous phase. In the pseudoternary phase diagram, the left area along the curve referred to turbid region, while the right was ME region. The dosage of vitamin D3 given to infants is relatively small, so there is a relatively large region in this pseudoternary phase diagram to form microemulsion. Meanwhile, the water content of saliva in the mouth is limited. Therefore, microemulsion can be formed by appropriately increasing the dosage of compound emulsifier.

### 3.2 Optimum formulation of SMEOFDF

In this study, an L9 (3^4^) orthogonal array was employed to evaluate the effect of three factors on formability and dissolution time of the films**.** The L9 (3^4^) orthogonal array corresponding to the nine rows and four columns is presented in Table 2, which consisted of nine experiments along with the experimental results and extreme difference analysis. In this matrix, the three selected factors were assigned to columns 1–3, and column 4 was designed to be empty. The analysis of variance (ANOVA) was performed by statistical software SPSS 12.0, shown in Table 3.

**Table 2:** Orthogonal experimental design and results

No.	Factors^a^				*Y*_1_ (point)	*Y*_2_ (s)	*Y* b
	A	B	C	e			
1	1	1	1	1	70	39.0	91
2	1	2	2	2	55	53.0	69
3	1	3	3	3	50	49.9	69
4	2	1	2	3	75	64.6	70
5	2	2	3	1	60	50.7	73
6	2	3	1	2	80	51.8	81
7	3	1	3	2	75	46.6	84
8	3	2	1	3	90	41.0	97
9	3	3	2	1	85	40.7	95
$\bar{I}$	76.27	81.41	89.64	86.40			
$\bar{\mathrm{II}}$	74.37	79.50	77.81	77.62			
$\bar{\mathrm{III}}$	91.96	81.71	75.16	78.58			
*R*	17.60	2.21	14.48	8.78			

a Empty column is denoted by e.

b Formability (*Y*_1_) takes 90 as the ideal value, and the weight is 40%. Dissolution time (*Y*_2_) takes 38 as the ideal value, and the weight is 60%. The calculation formula is below: $Y=40\times \frac{Y_{1}}{90}+60\times \frac{38}{Y_{2}}$.

**Table 3:** Analysis of variance

Source of variance	Sum of squares of deviation	*df*	Variance	*F*	Significance
A	186.47	2	93.24	7.57	*
B	2.87	2	1.44		
C	118.84	2	59.42		
e	46.36	2	23.18		
Error (B + e)	49.24	4	12.31		

* *P*<0.05.

*F*_0.05_ (2, 4) = 6.94.

Table 2 shows that the influence on the comprehensive weighted score decreased in order of A > C > B according to the *R* values. In the ANOVA (Table 3), the sum of squares of deviation (SSD) of columns 1–3 was calculated from different levels of factors A, B, and C. The SSD of the empty column (column 4) was also calculated. Obviously, this was not due to the different levels of the three factors selected, but to the experimental error. It was worth noting that the SSD of factor B (column 2) was less than the empty column (column 4), indicating that the factor B had no influence on experimental results. It may be assumed that SSD of factor B was also caused by experimental error in statistics. Therefore, the SSD of factor B (column 2) was pooled with the SSD of the empty column (column 4) to obtain the total SSD of experimental error (Liu, Xu, & Wang, 2012; Montgomery, 1997). Accordingly, the error accounts for four degrees of freedom, the total SSD of 49.24, and the variance of 12.31. In table *F*, *F*_0.05 _(2, 4) = 6.94, and *F* is less than *F* of factor A calculated in Table 3, demonstrating that factor A had a significant influence on formability and dissolution time. The level 3 of the major factor A should be chosen, while the levels 3 and 1 of the factors B and C could be chosen for better flexibility and formability of the films, respectively. In other words, optimum formulation of SMEOFDF was obtained when the MC dosage, glycerin dosage, and compound emulsifier dosage were 3 g, 12 ml, and 1 g in the 100 ml mixture, respectively. The duplicate tests with RSD 1.43% showed that the optimum formulation was reasonable.

### 3.3 Particle size, zeta potential, and morphology

**Figure 2:**
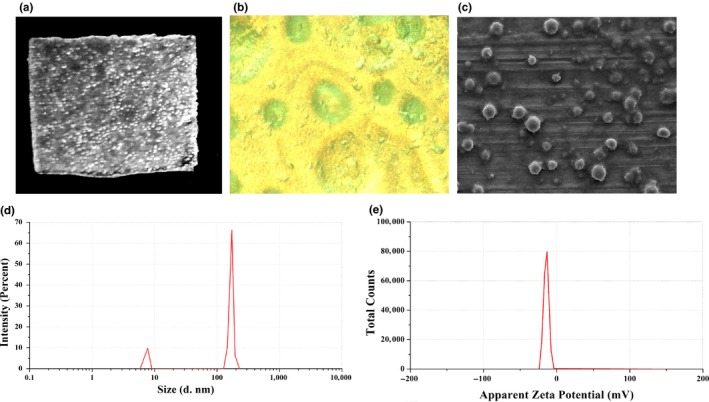
Investigation of particle size, zeta potential, and morphology. (a) SMEOFDF of vitamin D3 under sunlight, (b) microscopic characteristics of the surface under an inverted microscope, (c) morphology of redissolved emulsion under TEM, (d) representative figure of particle size determination, (e) representative figure of zeta potential determination

As shown in Figure 2a, the prepared SMEOFDF of vitamin D3 is a thin film, translucent, milky white under sunlight, with a uniform, smooth, and even appearance. It can be seen from Figure 2b that the microscopic characteristics of its surface were observed under an inverted microscope, and its surface was flat. The liquid self‐microemulsion was embedded in the dry films in the form of liquid drops, and the round, regular, and uniform pellets in the figure were microemulsion droplets. After SMEOFDF of vitamin D3 was dissolved, a drop of the solution was placed on a copper grid and observed under TEM. It can be seen that after this preparation was redissolved in water, uniform emulsion droplets could be formed (Figure 2c).

After SMEOFDF of vitamin D3 was redissolved in water, the particle size and zeta potential of the spontaneous microemulsion were shown in Figure 2d,e. The excipients used in these films are all soluble in water, so there will be no solid particles after dissolution. The average particle size measured is 181.2 nm, the multidispersion coefficient is 0.512, and the absolute value of potential is 16.1 mV, indicating that microemulsion has good stability and the particle size and potential meet the requirements.

### 3.4 Thickness, folding endurance, tensile strength, and elongation

The physical properties of SMEOFDF are shown in Table 4.

**Table 4:** Properties of SMEOFDF

Thickness (µm)	Tensile strength (MPa)	Elongation (%)	Folding endurance (time)	Disintegration time (s)
166.7 ± 3.30	38.45 ± 3.72	23.38 ± 4.23	>200	18 ± 1.23

The average thickness of the films was 166.7 ± 3.30 µm, indicating that the thickness was uniform and the weight difference was small. The test results of folding endurance, tensile strength, and elongation showed that the films had good toughness and tensile properties, which made it easy to be taken and stored. The disintegration time of the film was found less than 20 s and completely disappeared within 1.0 min, which was in line with the characteristics of rapid drug release.

### 3.5 In vitro release

**Figure 3:**
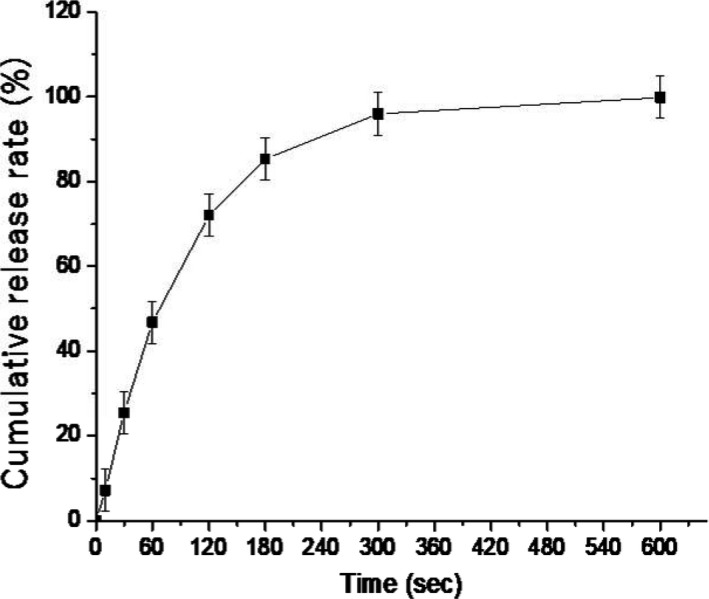
In vitro drug release curve of SMEOFDF

The average results of the test for in vitro release are shown in Figure 3 with *SD* bars. The vitamin D3 from SMEOFDF was quickly released at 37°C. The release amount of vitamin D3 increased with time, and its cumulative release rate was about 25% at 0.5 min, nearly 50% at 1 min, and more than 85% at 3 min.

In order to obtain meaningful information for release models, the release profiles were fitted to three different kinetic models, and the goodness of fit of the release data was assessed. Table 5 is a summary of the correlation coefficients for different release kinetic models for SMEOFDF of vitamin D3. Model with higher correlation coefficients was considered as a more appropriate model for the release data. As shown in Table 5, the best linear fitting parameter was the first‐order release model.

**Table 5:** Fitting of release results to different kinetic models

Kinetic models	*k*	*k*_0_	*M*_∞_	*r*
Zero‐order *M_t_*/*M*_∞_ = *k* × *t* + *k*_0_	0.0083	0.0067	100.0	0.9934
First‐order Ln (1−*M_t_*/*M*_∞_) = *k* × *t* + *k*_0_	−0.0107	0.0112	100.0	0.9959
Higuchi *M_t_*/*M*_∞_ = *k* × *t*^(1/2) + *k*_0_	0.0507	−0.0083	100.0	0.9730

## 4 CONCLUSIONS

A self‐microemulsifying oral fast dissolving films of vitamin D3 for infants was prepared in the present study. A ternary phase diagram was constructed to determine the concentration boundary of the microemulsion components in SMEOFDF. The film‐forming formula of SMEOFDF was screened by orthogonal experimental design. It was found that the MC dosage was a factor significantly affecting the formability and dissolution time of the films. SMEOFDF of vitamin D3 with a film‐like appearance had translucent milky white, smooth, and clean surface and can form microemulsion spontaneously after being redissolved in water. The film was of uniform thickness and good mechanical properties, and its disintegration time and cumulative release rate accorded with the characteristics of rapid drug release.

Self‐microemulsifying oral fast dissolving films of vitamin D3 can be taken by mouth directly, with convenient administration and accurate dosage. After entrance to the mouth, it dissolves quickly, tastes delicate, and spontaneously forms the microemulsion, which improves the dissolution rate and bioavailability, and has the advantages of good stability, easy to carry and preserve, and not to deteriorate. Different nutrients can be added according to the needs of different populations, so that they can be extended to all kinds of people. At the same time, the formulation and the production process are simple, suitable for industrial production, and the market prospect is good.

## CONFLICT OF INTEREST

The authors declare that they have no conflicts of interest.

## ETHICAL APPROVAL

The protocols and procedures were ethically reviewed and approved by Shanghai Ocean University. There was no human or animal testing in this study; ethics approval and consent to participate are not applicable to this manuscript.
